# Supplementary material for: SyNDI: synchronous network data integration framework
Source: BMC Bioinformatics. 2018 Nov 6;19:403. doi: 10.1186/s12859-018-2426-5 (PMC6219086; doi:10.1186/s12859-018-2426-5)
Supplement: Supplementary file 5 — Files of the example presented in Additional file 4. This additional file is a zip package that contains all files used in Additional file 4. Detailed descriptions of each of these files are presented in a README file included in this zip package. (GZ 30411 kb) [file 12859_2018_2426_MOESM5_ESM.gz › SyNDITools/Meme2Fimo/meme_4.9.0_4.tar.gz!/meme_4.9.0/doc/ama.xml]

AMA - a motif search tool


**Usage:**

`ama [options] <motif file> <sequence file> [<background file>]`

**Description:**

The name `AMA` stands for "Average Motif Affinity".
The program scores a set of DNA sequences given a DNA-binding motif,
treating each position in the sequence as a possible
binding event. The score is calculated by averaging the
likelihood ratio scores for all feasible binding events to
the given sequence and to its reverse strand.
The binding strength at each potential site is defined as
the likelihood ratio of the site under the motif versus under
a zero-order background model provided by the user.

By default, `AMA` reports the average motif affinity score.
It can also report *p*-values, which are estimated analytically
using the given zero-order background model or using the GC-content
of each sequence.

`AMA` can also compute the
sequence-dependent likelihood ratio score used by Clover. The
denominator of this score depends on the sequence being scored,
and is the likelihood of the site under a Markov model derived
from the sequence itself. Unlike Clover,
`AMA` also allows higher-order sequence-derived Markov
models (see *--sdbg* option below).

If the input file contains more than one motif, the motifs
will be processed consecutively.

Full details are given in the supplement to the GOMO paper:

Fabian A. Buske, Mikael Bóden, Denis C. Bauer and Timothy L. Bailey,
"Assigning
roles to DNA regulatory motifs using comparative genomics",
*Bioinformatics*, 26(7):860-866, 2010.

**Input:**

- `<motif file>` containing a list of motifs, in
  MEME format.
- `<sequence file>` is a collection of sequences in
  FASTA format.
- `[<background file>]` is a 0-order Markov model in
  background model format
  such as produced by
  `fasta-get-markov`.

**Output:**

`AMA` writes to standard out, unless you specify one of
`--o` or `--oc` in which case the `o-format`
option (if given) is ignored and separate files containing each output format are
written to the named directory. The available output formats are
gff and
CisML.

gff output has the format:

```
 <sequence_name> ama sequence 1 <sequence_length> <sequence_score> <sequence_p-value> . . .
```

**Options:**
